# Bimanual Reach to Grasp Movements in Youth With and Without Autism Spectrum Disorder

**DOI:** 10.3389/fpsyg.2018.02720

**Published:** 2019-01-17

**Authors:** Rachel A. Rodgers, Brittany G. Travers, Andrea H. Mason

**Affiliations:** ^1^Department of Kinesiology, University of Wisconsin–Madison, Madison, WI, United States; ^2^Occupational Therapy Program in the Department of Kinesiology, University of Wisconsin–Madison, Madison, WI, United States

**Keywords:** task complexity, age differences, prehension, kinematics, movement time, aperture

## Abstract

Reaching and grasping (prehension) is one of the earliest developing motor skills in humans, but continued prehension development in childhood and adolescence enables the performance of increasingly complex manual tasks. In individuals with autism spectrum disorder (ASD) atypical unimanual reaching and grasping has been reported, but to date, no studies have investigated discrete bimanual movements. We examined unimanual and bimanual reach to grasp tasks in youth with ASD to better understand how motor performance might change with increasing complexity. Twenty youth with ASD (10.1 ± 2.4 years) and 17 youth with typical development (TD) (9.6 ± 2.6 years) were instructed to reach and grasp cubes that became illuminated. Participants were asked to reach out with the right and/or left hands to grasp and lift targets located at near (18 cm) and/or far (28 cm) distances. For the unimanual (simplest) condition, participants grasped one illuminated cube (with either the left or right hand). For the bimanual conditions, participants grasped two illuminated cubes located at the same distance from the start position (bimanual symmetric condition) or two illuminated cubes located at different distances (bimanual asymmetric condition). Significant interactions among diagnostic group, task complexity, and age were found for initiation time (IT) and movement time (MT). Specifically, the older children in both groups initiated and performed their movements faster in the unimanual condition than in the bimanual conditions, although the older children with ASD produced slower ITs and MTs compared to typically developing peers across all three conditions. Surprisingly, the younger children with ASD had similar ITs and MTs as their peers for the unimanual condition but did not considerably slow these times to adjust for the complexity of the bimanual tasks. We hypothesize that they chose to re-use the motor plans that were generated for the unimanual trials rather than generate more appropriate motor plans for the bimanual tasks. An atypical spatiotemporal relationship between MT and peak aperture (PA) was also found in the ASD group. Together, our results suggest deficits in motor planning that result in subtle effects on performance in younger children with ASD that become more pronounced with age.

## Introduction

Autism spectrum disorder (ASD) is characterized by impairments in social communication, restricted interests, and/or repetitive behaviors (American Psychiatric Association [APA], 2013). In addition to these core symptoms, individuals on the autism spectrum demonstrate a diverse set of motor impairments (for reviews, see Fournier et al., 2010; Sacrey et al., 2014). Importantly, motor challenges in the first 2 years of life appear to be an early indicator of later ASD symptoms and a subsequent diagnosis of ASD (Bhat et al., 2012; Flanagan et al., 2012; LeBarton and Iverson, 2013; Libertus et al., 2014; Estes et al., 2015; Choi et al., 2018). Specifically, a delay in the development of fine motor skills between the ages of 6–24 months was predictive of which high-risk infants later received an ASD diagnosis (Landa and Garrett-Mayer, 2006; Landa et al., 2013; Choi et al., 2018). Libertus et al. (2014) further found that tasks related to grasping and object-exploration particularly distinguished infants at high risk and children at low risk for ASD. Therefore, reaching and grasping skills may be especially important to investigate in ASD, as poor reaching and grasping skills may detrimentally impact an infant’s ability to manually explore and learn about the world around them (Libertus et al., 2014).

While reaching and grasping (prehension) is one of the earliest developing motor skills in humans, this skill undergoes a prolonged developmental trajectory, becoming more and more refined through childhood and adolescence (Kuhtz-Buschbeck et al., 1998; Schneiberg et al., 2002; Smyth et al., 2004). Although no longitudinal studies have investigated the developmental trajectory of reaching and grasping in ASD during childhood and adulthood, case-control studies suggest that atypicalities in reach to grasp movements appear to persist past infancy. In studies using unimanual reach to grasp paradigms, slower MTs have been demonstrated in preschoolers with ASD (Campione et al., 2016) and school-aged children with ASD (Mari et al., 2003; Stoit et al., 2013). While this pattern of results is consistent with atypical motor execution other studies have found slower reaction times (RT) in youth with ASD when compared to TD peers for point to point aiming movements, suggesting deficits in motor planning (Glazebrook et al., 2006; Dowd et al., 2012). Additionally, studies have shown no group differences in grasp measures (Mari et al., 2003; Campione et al., 2016) nor how the participants adjusted their movements in response to objects of different sizes (Mari et al., 2003; Campione et al., 2016), different shapes (that afforded different grips) (Stoit et al., 2013), or different distances (Mari et al., 2003). Reach to drop paradigms that resemble reach to grasp paradigms have also found slower movement times (MTs) in children with ASD (Forti et al., 2011) (but see Fabbri-Destro et al., 2009 for an exception to this). Therefore, while some elements of movement may be similar in individuals with ASD and individuals with typical development, RT and MT have been shown to be significantly slower in ASD.

Taken together, the literature suggests that RT and MT may be key variables to distinguish reach to grasp performance in children with ASD and children with typical development. However, to date, only one-handed reach to grasp has been studied in ASD, and it is unclear how individuals with ASD perform bimanual reach to grasp tasks (i.e., reaching with the goal of grasping separate targets with two limbs). While no studies have investigated discrete bimanual reach to grasp in ASD, Piedimonte et al. (2018) recently reported the results of a study looking at continuous bimanual drawing in adolescents and adults with autism spectrum conditions compared to control participants. Their results indicated that there was a significant coupling effect between the limbs for both the ASD and control groups when they were asked to concurrently draw a circle with one hand and a line with the other. These results suggest similar bimanual coordination performance between the two groups. However, the authors also suggested that the circles-lines task may be a less demanding coordination task than reach-to-grasp since it involves mostly proximal muscles, and therefore may not be complex enough to elicit motor coordination differences (Piedimonte et al., 2018). The current study extends Piedimonte et al. (2018) by looking at kinematic performance in a discrete bimanual reach to grasp task. Bimanual reach-to-grasp also creates an interesting behavioral paradigm for studying motor development due to the challenges it places on the perceptual-motor control system (Bingham et al., 2008; Mason et al., 2010), as it is not possible to visually fixate both limbs and both targets at the same time when they are spatially separated. Therefore, it is necessary to sequentially divide visual attention between the reaching limbs and targets in order to successfully perform the task. The complexity of bimanual reach to grasp skills can be systematically manipulated by presenting participants with symmetric targets (i.e., both the same size or at the same distance from the start position) or asymmetric targets (i.e., targets of different sizes or at different locations) (Jackson et al., 1999). With task symmetry, spatial and temporal coordination of the two limbs is reinforced because the movements are similar between the two limbs. In contrast, with asymmetric tasks, spatial and temporal coordination is more challenging since the motor control system must coordinate two different reach-to-grasp programs. Studying bimanual reach to grasp in ASD is important, as many tasks in daily life require symmetric (i.e., grabbing pants at the waist band to dress or pulling a pan out of the oven) and asymmetric (i.e., putting on the sleeves of a shirt or grabbing a spatula while holding the handle of a frying pan) bimanual movements.

No studies have examined whether there are distinct age-related changes in reach to grasp performance in children with ASD compared to children with typical development. This is an important gap in our knowledge, as ASD is by definition a developmental disorder that persists throughout the life span and as other manual motor skills have been shown to have atypical developmental trajectories in ASD. Specifically, a longitudinal investigation found atypical developmental trajectories from childhood through mid-adulthood in ASD in finger tapping and grip strength measures (Travers et al., 2017), such that there was an atypically early plateau in these skills in the ASD group that led to more robust group differences in motor skills during adolescence and adulthood. A cross-sectional study similarly found more robust group differences in adolescence and adulthood in grip strength and finger tapping speed (Abu-Dahab et al., 2013), suggesting that motor deficits in ASD may become more pronounced over time compared to typically developing norms. If reach to grasp performance in unimanual and bimanual tasks show similar age-related changes, this would have implications for determining ideal time frames for interventions to address reach to grasp skills and their related daily living tasks.

The aim of the current study was to characterize diagnostic group differences in motor performance during reach to grasp under different levels of complexity (i.e., unimanual, bimanual symmetric, and bimanual asymmetric movements). Motor performance was operationally defined by the measures of initiation time (IT)^1^, MT, and peak grip aperture (PA). As a secondary aim, we were interested in determining whether task complexity interacted with age in children with ASD to further exacerbate motor deficits. Developmental changes in prehensile control are thought to be task-dependent. Specifically, Schneiberg et al. (2002) suggested that different aspects of movement kinematics mature at different rates, with movements requiring the coordination of a greater number of degrees of freedom taking longer to mature. Therefore, different rates of maturation may be evident when movements are produced by one limb (unimanual) or two limbs (bimanual), and the developmental trajectories may differ between typically developing youth and youth with ASD. Subsequently, we hypothesized that IT and MT in ASD would become increasingly slower compared to the typically developing group as both task complexity and age increased. Since no group differences between TD and ASD were found in PA in previous work, we were not expecting differences in the current experiment.

## Materials and Methods

### Participants

Participants were recruited through the Waisman Center’s participant registry and community fliers. Participants with ASD were included if they had a previous diagnosis of autistic disorder, Asperger’s syndrome, or pervasive developmental disorder not otherwise specified (PDD-NOS). Participants with ASD were excluded from this study if the family reported a known medical cause of ASD (i.e., fragile-X testing, tuberous sclerosis), hypoxia-ischemia, seizure disorder, or other neurological disorders. The Wechsler Abbreviated Scale of Intelligence (Wechsler, 1999) was performed to determine that participants did not have co-occurring intellectual disorder (full-scale IQ < 70). An autism diagnosis was confirmed using the Autism Diagnostic Observation Scale-2nd edition (ADOS-2) (Lord et al., 2012). However, two participants with a previous diagnosis of ASD narrowly missed cutoff on the ADOS-2. Because the results were equivalent with and without these two participants, we report results with these participants included.

All participants with typical development were required to have a score of less than eight on the Social Communication Questionnaire (SCQ) (Rutter et al., 2003). Further, participants with typical development were required to not have a first-degree family member with ASD, as motor difficulties may be present within the broader autism phenotype (Mosconi et al., 2010). All participants were verbal at the time of testing and had English as their first language.

Twenty-two participants with ASD and 21 participants with TD were recruited to participate in the study. Three individuals with typical development were excluded because they achieved a score of greater than eight on the SCQ, and three participants (two with ASD; one with TD) completed the reach to grasp paradigm, but their data were unusable because the motion capture LEDs were not visible at key kinematic landmarks (e.g., start or end of movement). As a result, the final sample included data from 20 children/adolescents with ASD and 17 children/adolescents with typical development (ages 6–16 years). Table 1 shows that participant groups were similar in age and performance IQ (PIQ), but the groups significantly differed in full-scale IQ (FSIQ) and verbal IQ (VIQ).

**Table 1:** Descriptive statistics for the demographic information in each group.

	ASD	Typically developing	
	(*n* = 20)	(*n* = 17)	*p*-Value
% Males	90.0%	82.4%	–
% Prefer right hand for writing	80.0%	94.1%	–
Age, Mean(SD)	10.14(2.39)	9.63(2.63)	0.55
Age, Range	6.36–14.49	6.37–16.47	–
FSIQ, Mean(SD)	102.45(12.78)	119.35(15.48)	0.001
FSIQ, Range	79–133	96–143	–
VIQ, Mean(SD)	100.47(12.10)	120.53(14.65)	<0.001
VIQ, Range	74–116	94–146	–
PIQ, Mean(SD)	104.42(17.82)	113.71(17.54)	0.12
PIQ, Range	71–143	87–145	–


*ASD, autism spectrum disorder; FSIQ, full-scale IQ; PIQ, performance IQ; VIQ, verbal IQ.*

This study received approval from University of Wisconsin–Madison’s institutional review board. Written informed consent was obtained from a parent/guardian, and assent was obtained from the children prior to data collection.

### Procedures

Participants were asked to complete three reach-to-grasp tasks. For the unimanual task they reached with either the right or left hand to grasp a lit plastic cube (2 cm × 2 cm × 2 cm) and transport it to a target receptacle. For the bimanual task they performed two synchronous one-handed reaches to grasp and lift two lit plastic cubes – one located to the left of the midline and one located to the right of the midline. The bimanual task consisted of symmetric and asymmetric conditions.

**FIGURE 1:**
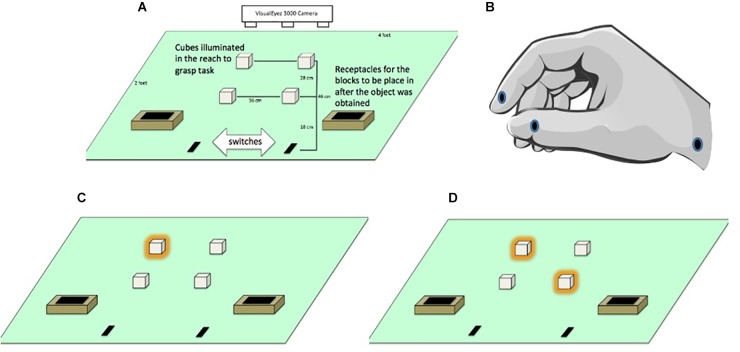
**(A)** General layout of apparatus monitored by a VisualEyez 3000 Camera. Participants reached forward to grasp and relocate translucent cubes (2 cm × 2 cm × 2 cm) lit by LEDs into “treasure chests” as quickly and as accurately as possible. **(B)** Position of the LEDs located on the index finger, thumb and wrist. **(C)** Illuminated targets in the unimanual left far condition. **(D)** Illuminated targets in the bimanual asymmetric left far/right near condition.

Participants were seated on a height-adjustable chair so their forearms were at the same level as the table when they produced an angle of ∼90° by flexing their elbows. They began the task with their index fingers and thumbs lightly touching while resting on start switches that were embedded into a rectangular table (see Figure 1A). The switches were located 3.5 cm from the edge of the table in front of the subjects and served as the start and end positions for the right and left hands. Participants were asked to keep these switches depressed at all times, unless they were reaching for or transporting the cubes.

Since we worked with children we referred to the hand position for reaching and grasping the cubes as “crab pinchers” to remind them to keep their index finger and thumb in pad opposition to perform each movement (see Figure 1B). The experimenter initiated each trial by lighting one or two cubes. Participants were instructed to reach from the start position to grasp the cube(s) and transported them to treasure chests located near the start position (see Figures 1C,D). Participants were asked to move as quickly and accurately as possible, but to make sure not to miss, bump, or drop the cube. Once they grasped the target(s) they were asked to transport them to the treasure chests. To maintain motivation throughout the experiment, participants were told that they were placing cubes into a treasure chest and their progress through the trials was marked on a whiteboard as if it was a board game.

Infrared LEDs (light emitting diodes) were attached to the distal lower corner of the index fingers, distal lower corner of the thumbnails and styloid process of the wrists using medical tape (see Figure 1B). Kinematic data were recorded for the participants’ hand movements using a VisualEyez 3000 (Phoenix Technologies Inc., Burnaby, British Columbia) three-dimensional motion capture system mounted above the table to monitor the position of the LEDs. Participants were asked to grasp cubes located on the right side of the table only with the right hand and cubes on the left side of the table only with the left hand.

Data collection began with a practice set consisting of 10 unimanual trials followed by 4 bimanual trials. We then verbally confirmed that the participant was ready to begin. Each participant then performed reach-to-grasp movements in two unimanual and four bimanual conditions. For the unimanual conditions participants grasped the lit cubes with either the right or left hand at either a near (18 cm) or far (28 cm) position. They performed eight unimanual trials per condition for a total of 32 randomized trials. During the bimanual symmetric conditions, participants performed reach-to-grasp movements with both hands toward both near or both far cubes while the bimanual asymmetric conditions involved reaching for a near and a far cube. They performed a total of 56 randomized bimanual trials. The total protocol took 30–45 min.

### Data Recording and Processing

Position data from the LEDs were sampled at 200 Hz (RMSE = 0.70 mm). For each reach to grasp movement, the 3-D spatial coordinates of LEDs were stored, and then analyzed off-line using customized software (KinSys, EZSoft, Madison, WI, United States). The position data were first interpolated over missing data points of no more than four frames (20 ms) and then low-pass filtered at a cutoff frequency of 7 Hz using a bi-directional Butterworth filter. Trials were discarded when more than 4 consecutive frames of missing data occurred within the movement (1.9% of unimanual trials, 2.5% of bimanual symmetric trials, and 2.6% of bimanual asymmetric trials). Trials in which the participant grasped the wrong cube or grasped the correct cube with the wrong hand were marked as inaccurate and were also omitted from MT calculations. Overall, there was 97% accuracy in the ASD group and 98% accuracy in the TD group, which did not statistically differ, *t*(35) = -1.11, *p* = 0.28. An algorithm followed by visual verification of wrist velocity profiles was used to determine start and end of movement. To determine start of movement (IT) the algorithm searched backward from the peak resultant wrist velocity for the first appearance of a velocity less than 5 mm/s. End of movement was defined as the minimum value (valley) between the peaks defining the initial reach to grasp and object transport to treasure chest with object lift determined to occur if velocity increased above 110 mm/s within 10 frames. Start and end of movement were determined using the wrist LED. To quantify grasp formation, the magnitude of the position difference between the LEDs on the index finger and thumb was computed at each frame. Peak aperture was then identified for each trial. While multiple kinematic measures can be derived from these data, these analyses examine performance using IT in milliseconds, MT in milliseconds and PA in millimeters. MT and PA are common kinematic measures for analyzing movement coordination and planning and provide behavioral information about the role of task complexity in movement planning and performance.

### Data Analysis

All statistical analyses were performed in *R* version 3.4.4 (R Core Team, 2018). Using the lmerTest package (Kuznetsova et al., 2017), we employed hierarchical linear modeling (HLM) in order to simultaneously consider variability within and between participants, as per our study design. Three separate HLMs were performed: the first used median IT as the dependent variable, the second used median MT as the dependent variable, and the third used mean PA as the dependent variable. Medians were used for the response time variables to prevent outliers from skewing the averages. For all three HLMs, the within-subject (Level 1) predictors were distance (near reach versus far reach) and hand (left hand versus right hand). The between-subject (Level 2) predictors were age (years, mean-centered to reduce the chance o multicollinearity), diagnostic group (ASD versus typical development), and condition (unimanual reach, bimanual symmetric reach [both hands reaching for near targets or both hands engaging reaching for far targets], and bimanual asymmetric reach [one hand reaching for a near target and one hand reaching for a far target]). Analyses were performed both with and without IQ as a covariate. However, IQ did not improve the model (as assessed by AIC [Akaike, 1974]), and the same pattern of results emerged when including IQ or not. Therefore, the results are reported without IQ in the model.

For all analyses, *p*-values for fixed effects were calculated using an Analysis of Variance Table of type III with Satterthwaite approximation for degrees of freedom, and an alpha of 0.05 was used to assess statistical significance.

## Results

**FIGURE 2:**
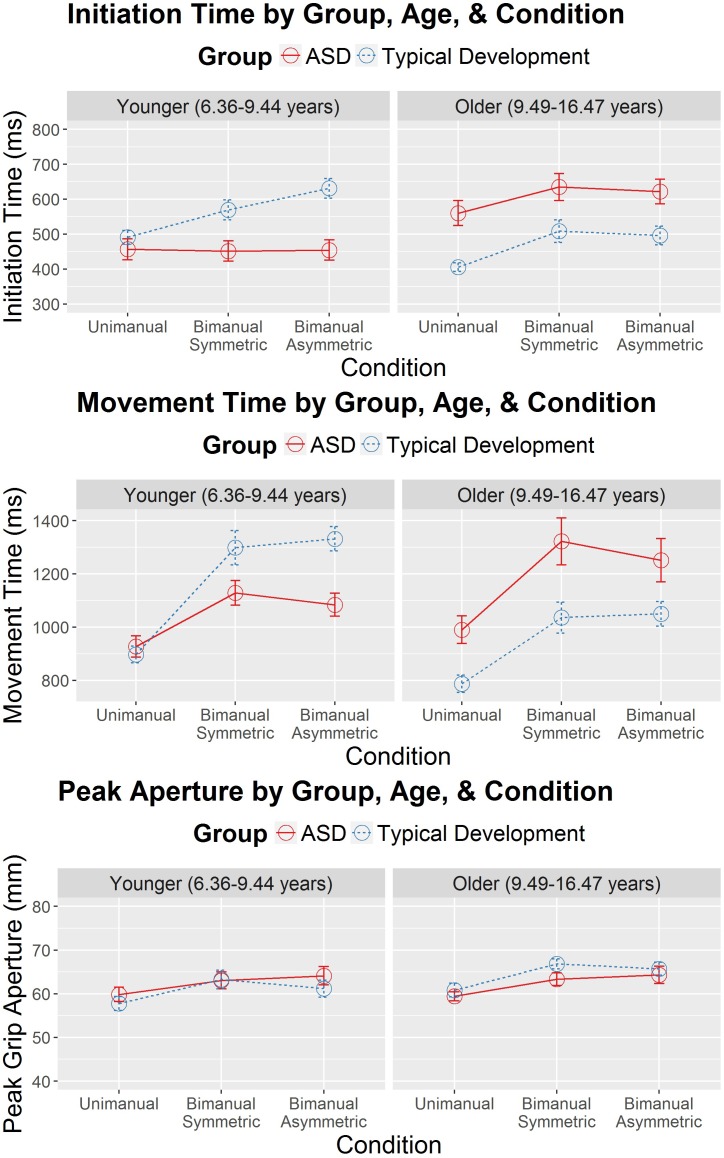
Initiation time (in milliseconds) and movement time (in milliseconds) as a function of diagnostic group (autism spectrum disorder [ASD] and typical development), age (years), and condition (unimanual, bimanual symmetric, and bimanual asymmetric). Error bars represent the standard error. Age was treated as a continuous variable in the hierarchical linear model (HLM). However, to simplify the presentation, a median split (median = 9.44 years) was used to separate the younger and older participants.

All assumptions of HLM were checked and found to be met for the analysis of IT, MT, and PA. Descriptive data for these analyses appear in Supplementary Tables 1–3 and the fixed effects are shown in Table 2. The only significant effect for PA was for condition, suggesting that task complexity affected PA across the participants. However, for IT and MT, there was a significant three-way interaction among diagnostic group, condition, and age. These three-way interactions are illustrated in Figure 2. While the HLM treated age as a continuous variable for analysis, Figure 2 simplifies this interaction by showing the pattern of IT and MT across conditions in the different diagnostic groups with a median split of age (“Younger” ≤ 9.44 years; “Older” > 9.44 years). The three-way interaction suggests that pattern of IT and MT among the groups differed as a function of age and task complexity.

**Table 2:** Results for fixed effects of hierarchical linear model (HLM) assessing initiation time (medians), movement times (medians), and peak grip aperture as a function of group (ASD versus typical development), age (years), and condition (unimanual reaching, bimanual symmetric reaching, and bimanual asymmetric reaching).

	Sum of squares	Mean square	*df*	*F*	*p*-Value
		**Initiation time (IT)**			
Group	1362.13	1362.13	1,33.03	0.09	0.76
**Condition**	**396356**.**28**	**198178**.**14**	**2,397**.**43**	**13**.**23**	**<0**.**001**
Age	8726.60	8726.60	1,33.03	0.58	0.45
**Group** × **condition**	**158291**.**87**	**79145**.**94**	**2,397**.**43**	**5**.**28**	**0**.**005**
**Group** × **age**	**181018**.**17**	**181018**.**17**	**1,33**.**03**	**12**.**09**	**0**.**001**
**Condition** × **age**	**151517**.**74**	**75758**.**87**	**2,397**.**43**	**5**.**06**	**0**.**007**
**Group** × **condition** × **age**	**209318**.**71**	**104659**.**36**	**2,397**.**43**	**6**.**99**	**0**.**001**
		**Movement time (MT)**			
Group	481.57	481.57	1,33.00	0.01	0.92
**Condition**	**7635410**.**52**	**3817705**.**26**	**2,357**.**02**	**79**.**50**	**<0**.**001**
Age	67.03	67.03	1,33.00	<0.01	0.97
**Group** × **condition**	**385705**.**88**	**192852**.**94**	**2,357**.**02**	**4**.**02**	**0**.**02**
**Group** × **age**	**200411**.**15**	**200411**.**15**	**1,33**.**00**	**4**.**17**	**<0**.**05**
Condition × age	17307.13	8653.57	2,357.02	0.18	0.84
**Group** × **condition** × **age**	**820811**.**02**	**410405**.**51**	**2,357**.**02**	**8**.**55**	**<0**.**001**
		**Peak grip aperture (PA)**			
Group	8.36	8.36	1,33.00	0.24	0.63
**Condition**	**2056**.**38**	**1028**.**19**	**2,361**.**00**	**29**.**47**	**<0**.**001**
Age	39.48	39.48	1,33.00	1.13	0.30
Group × condition	146.27	73.14	2,361.00	2.10	0.12
Group × age	1.31	1.31	1,33.00	0.04	0.85
Condition × age	1.42	0.71	2,361.00	0.02	0.98
Group × condition × age	128.78	64.39	2,361.00	1.85	0.16


*Random effects for this model included participant (as these were repeated measurements), whether the right or left hand movement time was measured, and whether the reach was near or far. Significant effects are bolded. df, degrees of freedom.*

Significant interactions between group and condition were also present for IT and MT, suggesting that groups showed different patterns of IT and MT across the different reach-to-grasp conditions. Figure 2 suggests that IT and MT varied more among the different reach-to-grasp conditions in the TD group than in the ASD group. Follow-up HLMs in each group demonstrated that in the TD group task complexity affected IT, *F*(2,165.23) = 21.27, *p* < 0.001, and MT, *F*(2,181.67) = 76.54, *p* < 0.001. However, task complexity did not affect IT in the ASD group, *F*(2,215.10) = 0.93, *p* = 0.40, and while MT varied as a function of condition in the ASD group, *F*(2,197.06) = 23.96, *p* < 0.001, the group-by-condition interaction suggested that this was to a lesser degree than in the TD group. Therefore, while the TD group showed ITs and MTs that modulated as a function of task complexity, the ASD group’s ITs and MTs showed diminished modulation according to task complexity.

Because the overall HLMs for IT and MT suggested a significant interaction between diagnostic group and age, follow-up analyses were performed to examine IT and MT as a function of the different ages of children in the study and diagnostic group status. Since we were interested in evidence of different age-related, cross-sectional changes in IT and MT as a function of diagnostic group status, only the interaction effects were interpreted. These analyses were performed separately for unimanual, bimanual symmetric, and bimanual asymmetric reaches and the figures for these can be seen in Supplementary Figure 1. Representative hand velocity profiles are also shown in Supplementary Figures 2, 3 for one younger child with TD, one younger child with ASD, one older child with TD and one older child with ASD. For unimanual reaches, there was no interaction between age and diagnostic group for MT, *F*(1,33.00) = 1.35, *p* = 0.25. However, there was a significant interaction for unimanual reaches between age and diagnostic group for IT, *F*(1,34.81) = 5.41, *p* = 0.03. Significant age-by-group interactions appeared for bimanual symmetric reaches in IT, *F*(1,33.04) = 9.97, *p* = 0.003, but not for MT, *F*(1,33.00) = 34.57, *p* = 0.07. Significant interactions also appeared for bimanual asymmetric reaches in IT, *F*(1,33.02) = 12.44, *p* = 0.001, and MT, *F*(1,33.51) = 5.32, *p* = 0.03. In summary, older individuals with TD had faster ITs than younger individuals with TD, indicative of faster ITs with age. In contrast, older individuals with ASD tended to have slower ITs than younger participants with ASD, an age trend which was significantly distinct from that of the TD group across all three levels of complexity. MT followed the same pattern of results, although these age effects were only statistically different between groups in the bimanual asymmetric condition.

**FIGURE 3:**
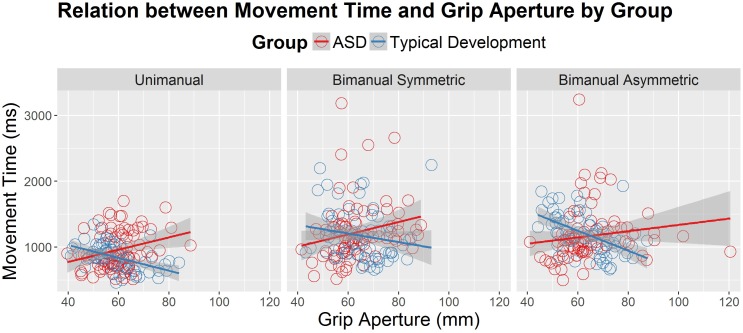
Movement time (in milliseconds) as a correlate of peak grip aperture (mm), age (years) and diagnostic group (autism spectrum disorder [ASD] and typical development), across the different conditions (unimanual, bimanual symmetric, and bimanual asymmetric). There was a significant interaction between diagnostic group and peak aperture, such that the typically developing group demonstrated the expected negative relations between grip aperture and movement time, whereas the ASD group demonstrated positive relations between grip aperture and movement time.

Since MTs tended to be shorter for the younger group with ASD compared to TD, a follow-up HLM analysis was performed to examine whether PA was associated with MT. Specifically, we were interested in determining whether participants who used shorter MTs also used a wider grip aperture to compensate for those shorter MTs. The relation between MT and PA is shown Figure 3 for the three conditions and two groups (ASD and TD). Table 3 reports the fixed effects of the HLM predicting MT as a function of PA, group, condition, and age. In addition to the three-way interaction among group, condition, and age that was already reported for MT above, there was a significant two-way interaction between group and PA. Typically developing children in both age groups demonstrated the expected negative relationship between MT and PA, such that PAs were larger for shorter MTs. In contrast, youth with ASD demonstrated the opposite effects: as MTs got shorter, PAs got smaller. The lack of other significant interactions suggested that the atypical relation between PA and MT in the ASD group compared to the TD group persisted regardless of task condition and age.

**Table 3:** Results for fixed effects of hierarchical linear model (HLM) assessing movement times (medians) as a function of peak grip aperture, group (ASD versus typical development), age (years), and condition (unimanual reaching, bimanual symmetric reaching, and bimanual asymmetric reaching).

	Sums of squares	Mean square	*df*	*F*	*p*-Value
Group	455.27	455.27	1,30.75	0.01	0.92
**Condition**	**5531967**.**60**	**2765983**.**80**	**2,353**.**95**	**61**.**09**	**<0**.**001**
Peak grip aperture	23063.35	23063.35	1,413.09	0.51	0.48
Age	1675.82	1675.82	1,31.31	0.04	0.85
Group × condition	154300.73	77150.36	2,353.54	1.70	0.18
**Group** × **peak grip aperture**	**522473**.**92**	**522473**.**92**	**1,413**.**57**	**11**.**54**	**0**.**001**
Condition × peak grip aperture	47375.07	23687.53	2,352.11	0.52	0.59
Group × age	169840.87	169840.87	1,31.30	3.75	0.06
Condition × age	10803.23	5401.62	2,355.01	0.12	0.89
Peak grip aperture × age	29401.75	29401.75	1,406.72	0.65	0.42
Group × condition × peak grip aperture	70571.34	35285.67	2,352.30	0.78	0.46
**Group** × **condition** × **age**	**564869**.**28**	**282434**.**64**	**2,355**.**05**	**6**.**24**	**0**.**002**
Group × peak grip aperture × age	6609.29	6609.29	1,406.60	0.15	0.70
Condition × peak grip aperture × age	86720.78	43360.39	2,352.23	0.96	0.38
Group × condition × peak grip aperture × age	27120.49	13560.24	1,352.22	0.30	0.74


*Random effects for this model included participant (as these were repeated measurements), whether the right or left hand movement time was measured, and whether the reach was near or far. Significant effects are bolded. df, degrees of freedom.*

## Discussion

The purpose of this study was to compare reach-to-grasp movements in youth on the autism spectrum and youth with typical development across increasingly more challenging conditions (i.e., unimanual, bimanual symmetric, and bimanual asymmetric reaches) and to examine potential age effects across our cross-sectional cohort. While typically developing children generally improve in their motor performances as they age (see Haywood and Getchell, 2014), there may be different developmental trajectories for simple versus complex motor skills across the lifespan in children and youth with ASD, particularly as tasks become more complex and learning curves come into play. The findings from the current study underscore that participant age and task complexity are important moderators for the planning and performance of reach-to-grasp movements in children with ASD.

Specifically, older children (∼9.5 years and older) with ASD showed slower movement (both ITs and MTs) compared to typically developing peers across all three levels of task complexity. This is consistent with Glazebrook et al. (2006) who found longer MTs for an aiming task in participants with ASD (mean age 25.1 years) when compared to TD peers and Stoit et al. (2013) who also found slower MTs for a dowel grasping task in youth with ASD (mean age 11.6 years) when compared to TD peers. In contrast, in the current study, the younger individuals with ASD showed no difference from typically developing peers in ITs or MTs in the simplest (unimanual) task, and actually produced faster movement (both IT and MT) compared to typically developing peers in the more complicated bimanual tasks. While surprising that younger children with ASD may outperform age-matched children with typical development, these results are not unprecedented. Recent studies have reported similar or enhanced motor performance at younger ages in ASD compared to TD for simple tasks such as drawing a line between two targets (Papadopoulos et al., 2012), using a stylus to perform point to point movements (Dowd et al., 2012) or reaching to grasp a single target object (Fabbri-Destro et al., 2009). Together, these results suggest a pattern of similar or enhanced motor performance at younger ages in ASD compared to TD but more impaired motor performance at older ages in ASD compared to TD) (Travers et al., 2017). An advantage of the present study is the combination of temporal and spatial information about the reach to grasp movement, which may help explain factors underlying the atypical development of motor skills in ASD.

One potential explanation for the enhanced performance in younger children but poorer performance in older children with ASD is that motor challenges are not present in younger children with ASD. Instead these motor challenges appear and become more conspicuous in later childhood and adolescence. This explanation is unlikely because it is counter to reports from medical records (Ming et al., 2007) and literature documenting motor differences in very young children on the autism spectrum (Bhat et al., 2012; Flanagan et al., 2012; LeBarton and Iverson, 2013; Libertus et al., 2014; Estes et al., 2015; Choi et al., 2018).

Another potential explanation is that younger children with ASD may not modulate their ITs or MTs as a function of task complexity. The present results are in support of this, as the younger ASD group demonstrated no statistically significant difference in ITs across the increasingly complex task conditions, whereas the TD group did. However, both the young ASD and TD groups demonstrated MTs that were modulated as a function of task complexity, although the modulation was not as large in the ASD group. These results suggest that task complexity affects both motor planning and movement execution in typical development, replicating previous research on discrete bimanual tasks in both adults and typically developing youth (Jackson et al., 1999; Bingham et al., 2008; Mason et al., 2010, 2013). However, task complexity may affect movement execution but not motor planning in young children with ASD. In other words, younger children with ASD may re-use the same motor plans across easy and more complicated tasks, resulting in similar ITs regardless of task complexity. These results support recent evidence by Studenka and Cummins (2017) who documented a resistance to formulating new motor plans in children with ASD. Specifically, they found that children with ASD persisted in using previous motor plans even when new plans would result in more efficient or comfortable movement. Studenka and Cummins (2017) suggested that, for individuals with ASD, maintaining and using a previous motor plan represents a smaller “motor planning cost” than avoiding an uncomfortable posture. Since movement planning requires both choosing the endpoint of the movement and selecting an appropriate motor program to bring the arm and hand to that endpoint, optimal control would suggest that movement plans are updated on a trial-by-trial basis to take into account the constraints of the current task (i.e., target location, size, etc.). However, in order to achieve this optimal control, there are also switching costs associated with abandoning an old motor plan in favor of a new, more appropriate plan (Orban de Xivry and Lefevre, 2016). The current data, along with the work of Studenka and Cummins (2017) suggests the younger children with ASD may have increased resistance to motor plan switching.

As feedback becomes available during movement execution, younger children with ASD can make use of that feedback to modify the movement plan in the more complex bimanual conditions. However, the fact that the MTs produced by the young children with ASD did not slow to the levels seen in TD children, suggests that movement execution is also not typical. As suggested by previous studies, these atypicalities in movement execution may be the result of deficits in the use of online sensory feedback (Glazebrook et al., 2009; Mosconi et al., 2015).

In the current study, re-using the same motor plans across task complexity conditions may have benefited the younger children with ASD as they achieved faster ITs and MTs and did not produce more task completion errors. However, there are likely tasks where re-use of the motor plan and atypical motor execution may not confer a benefit but may instead result in significant performance deficits. If the task was made substantially more complex and the young children with ASD continued to use a strategy of not slowing down (or opening their hand wider), then we may see more obvious performance errors. For example, a more complex bimanual task that required grasping fragile objects, tippy objects, or objects full of liquid would require significant slowing of the movements in order to be successful. If the younger group with ASD continued to use faster MTs in these conditions, then the consequences of not adjusting would be much more significant. To our knowledge, the present study is the first to systematically affect task complexity in a bimanual reach to grasp paradigm in ASD. Therefore, future investigations are needed to confirm and expand upon this possibility.

A third potential explanation for the IT and MT results seen in the younger ASD group is that youth with ASD may be coordinating speed of movement and spatial elements of movement (i.e., spatiotemporal coordination) differently than youth with typical development. In this study, peak grip aperture was similar in both groups regardless of task complexity and age. However, the relations between MT and PA across the three task conditions were different in the ASD group compared to the TD group. Specifically, faster movements to the target (i.e., shorter MTs) were not accompanied by larger PAs in ASD, as would be expected from the adult TD literature (Wing et al., 1986; Wallace and Weeks, 1988) and from the relations observed in the current experiment in the TD group. In contrast, faster movements to the target were associated with smaller grips in the ASD group, which was observed across all three task conditions and regardless of age. Because this pattern was a commonality across all levels of complexity and all ages, it is possible that the current pattern of results are due to the ASD group not widening the hand grip to accommodate for the lack of spatial accuracy that is often accompanied by faster movements. Wing et al. (1986) suggested that PA is planned in advance of movement execution using internalized information about the predicted accuracy of the transport component. The accuracy of the transport component decreases as movement speed increases (Fitts, 1954). Therefore, larger PAs are planned and executed when performers predict greater reaching errors based on past experience. The atypical relationship between the speed of transport and the formation of the grasp in ASD may therefore provide additional evidence of atypical planning processes in ASD. Further, these results may suggest that planning atypicalities are related to deficits in the effective internalization of transport error information over several trials (Wing et al., 1986; Mostofsky and Ewen, 2011). When considering the implications of this atypical pattern of coordination for activities of daily living, these results suggest that deficits in skills such as catching a ball (Manjiviona and Prior, 1995; Ament et al., 2015) could be related to poor spatiotemporal planning for the coordination of arm transport with hand grip formation.

### Limitations

One potential limitation of the current study is the ordering of the tasks. Participants completed all unimanual trials before moving on to the bimanual trials, which may limit our ability to determine whether the same motor plan is being used in both conditions. We chose to have our participants complete all unimanual trials before the bimanual trials for two reasons. First, we felt that it was important to progress task difficulty by starting with simple unimanual tasks before proceeding to the more difficult bimanual tasks. Second, for the unimanual trials to be considered true unimanual control trials, we felt that it was important that the movement planning processes not include preparation for both unimanual and bimanual performance. Future work is needed to assess whether being exposed to the bimanual conditions first or completely randomizing all conditions has a significant effect on movement planning and performance.

## Conclusion

Many activities of daily living require us to reach for, grasp, and manipulate the objects in our surroundings. Many of these activities also require the simultaneous use of both hands. The purpose of this study was to determine whether the added complexity of planning, executing and monitoring the movements of two hands would be differentially more difficult for individuals with ASD and whether age-related changes in motor performance would be moderated by task complexity. Our results indicated that younger children with ASD did not adjust their movements in a typical way when presented with a more complex bimanual skill. They used the same ITs across conditions and did not modulate their MTs as much as their TD peers. In contrast, the older ASD group had slower ITs and MTs when compared to their TD peers across conditions. Although these results appear to suggest a transition from better performance in younger children with ASD to worse performance with increasing age, it is also important to consider the atypical spatiotemporal relationship between MT and PA found in the ASD group when compared to their TD peers, regardless of condition or age. These results highlight the need to not only consider kinematic variables such as IT or MT in isolation, but to also consider how these variables are coordinated in time and space with other important kinematic variables such as PA. In particular, for studies that have shown similar or better performance in children with ASD compared to TD peers earlier in life but poorer performance later in life, the use of IT or MT as a dependent variable without considering measures of movement coordination may have led to incomplete interpretations. Depending on the measure used to operationally define motor performance, subtle motor challenges (like atypical motor planning) might be masked in ASD early in life.

## Author Contributions

AM and BT contributed to the conception and design of the study. RR collected and analyzed the kinematic data. BT performed the statistical analysis. RR, BT, and AM wrote the sections of the manuscript. All authors contributed to manuscript revision, read and approved the submitted version.

## Conflict of Interest Statement

The authors declare that the research was conducted in the absence of any commercial or financial relationships that could be construed as a potential conflict of interest.

## Footnotes

1 Initiation time was measured as the time between the cue (illuminated block or blocks) and the start of movement. We have chosen the term “initiation time” rather than “reaction time” because unlike traditional reaction time paradigms, participants were not instructed to see how quickly they could grasp the target after the cue. Instead, we encouraged participants to reach to grasp the target as quickly and as accurately as possible.
