# Supplementary material for: Bimanual Reach to Grasp Movements in Youth With and Without Autism Spectrum Disorder
Source: Front Psychol. 2019 Jan 17;9:2720. doi: 10.3389/fpsyg.2018.02720 (PMC6344405; doi:10.3389/fpsyg.2018.02720)
Supplement: Supplementary file 1 [file Data_Sheet_1.docx]

**Supplemental Table 1.** Descriptive statistics for HLM model predicting Initiation Time (IT) in ms. Hand and distance were used as random effects in the hierarchical linear model. Age effects are not shown here.

|  |  |  | **ASD Initiation Time (ms)** | | **Typically Developing Initiation Time (ms)** | |
| --- | --- | --- | --- | --- | --- | --- |
| **Condition** | **Hand** | **Distance** | **Mean** | **SD** | **Mean** | **SD** |
| Unimanual | Left | Near | 506 | 247 | 449 | 104 |
|  | Right | Near | 462 | 139 | 426 | 93 |
|  | Left | Far | 531 | 233 | 490 | 105 |
|  | Right | Far | 536 | 231 | 439 | 121 |
|  |  | **Total** | **508** | **215** | **451** | **106** |
| Bimanual Symmetric | Left | Near | 528 | 209 | 513 | 163 |
|  | Right | Near | 540 | 202 | 477 | 138 |
|  | Left | Far | 551 | 278 | 600 | 190 |
|  | Right | Far | 555 | 255 | 574 | 206 |
|  |  | **Total** | **543** | **234** | **541** | **179** |
| Bimanual Asymmetric | Left | Near | 510 | 201 | 557 | 173 |
|  | Right | Near | 544 | 233 | 569 | 159 |
|  | Left | Far | 554 | 241 | 588 | 172 |
|  | Right | Far | 543 | 216 | 556 | 203 |
|  |  | **Total** | **538** | **220** | **568** | **174** |

**Supplemental Table 2.** Descriptive statistics for HLM model predicting Movement Time (MT) in ms. Hand and distance were used as random effects in the hierarchical linear model. Age effects are not shown here.

|  |  |  | **ASD Movement Time (ms)** | | **Typically Developing Movement Time (ms)** | |
| --- | --- | --- | --- | --- | --- | --- |
| **Condition** | **Hand** | **Distance** | **Mean** | **SD** | **Mean** | **SD** |
| Unimanual | Left | Near | 861 | 283 | 810 | 190 |
|  | Right | Near | 880 | 290 | 748 | 180 |
|  | Left | Far | 1077 | 287 | 982 | 177 |
|  | Right | Far | 1019 | 273 | 841 | 162 |
|  |  | **Total** | **959** | **293** | **845** | **194** |
| Bimanual Symmetric | Left | Near | 1085 | 283 | 1073 | 327 |
|  | Right | Near | 1115 | 398 | 1040 | 322 |
|  | Left | Far | 1308 | 447 | 1312 | 398 |
|  | Right | Far | 1394 | 585 | 1275 | 425 |
|  |  | **Total** | **1225** | **453** | **1175** | **382** |
| Bimanual Asymmetric | Left | Near | 1073 | 277 | 1236 | 338 |
|  | Right | Near | 1324 | 593 | 1229 | 306 |
|  | Left | Far | 1220 | 379 | 1185 | 260 |
|  | Right | Far | 1054 | 313 | 1146 | 322 |
|  |  | **Total** | **1168** | **416** | **1199** | **303** |

**Supplemental Table 3.** Descriptive statistics for HLM model predicting Peak Grip Aperture (PA) in mm. Hand and distance were used as random effects in the hierarchical linear model. Age effects are not shown here.

|  |  |  | **ASD Peak Grip Aperture (mm)** | | **Typically Developing Peak Grip Aperture (mm)** | |
| --- | --- | --- | --- | --- | --- | --- |
| **Condition** | **Hand** | **Distance** | **Mean** | **SD** | **Mean** | **SD** |
| Unimanual | Left | Near | 60.14 | 9.89 | 59.95 | 9.65 |
|  | Right | Near | 60.41 | 10.13 | 59.55 | 10.46 |
|  | Left | Far | 58.89 | 6.18 | 59.23 | 7.93 |
|  | Right | Far | 59.16 | 8.42 | 58.14 | 9.71 |
|  |  | **Total** | **59.65** | **8.65** | **59.22** | **9.29** |
| Bimanual Symmetric | Left | Near | 64.11 | 11.52 | 66.13 | 9.16 |
|  | Right | Near | 63.58 | 10.38 | 63.77 | 11.78 |
|  | Left | Far | 62.55 | 10.32 | 64.60 | 9.02 |
|  | Right | Far | 62.58 | 11.26 | 65.37 | 12.31 |
|  |  | **Total** | **63.20** | **10.70** | **64.97** | **10.47** |
| Bimanual Asymmetric | Left | Near | 65.38 | 11.13 | 67.01 | 10.24 |
|  | Right | Near | 65.07 | 12.66 | 64.28 | 12.14 |
|  | Left | Far | 61.10 | 10.13 | 61.04 | 8.72 |
|  | Right | Far | 65.36 | 16.87 | 60.89 | 10.47 |
|  |  | **Total** | **64.23** | **12.84** | **63.31** | **10.54** |
