# Supplementary material for: Bimanual Reach to Grasp Movements in Youth With and Without Autism Spectrum Disorder
Source: Front Psychol. 2019 Jan 17;9:2720. doi: 10.3389/fpsyg.2018.02720 (PMC6344405; doi:10.3389/fpsyg.2018.02720)
Supplement: Supplementary file 2 [file Data_Sheet_2.docx]

**Supplemental Figure 1.** Initiation time, movement time, and peak grip aperture as a correlate of age (years) and diagnostic group (autism spectrum disorder [ASD] and typical development), across the different conditions. There were significant interactions between age and diagnostic group in initiation time and movement time but not in peak grip aperture, suggesting faster performance in older participants with typical development but slower performance in older participants with ASD. In contrast, there were no age-by-group interactions in grip aperture.
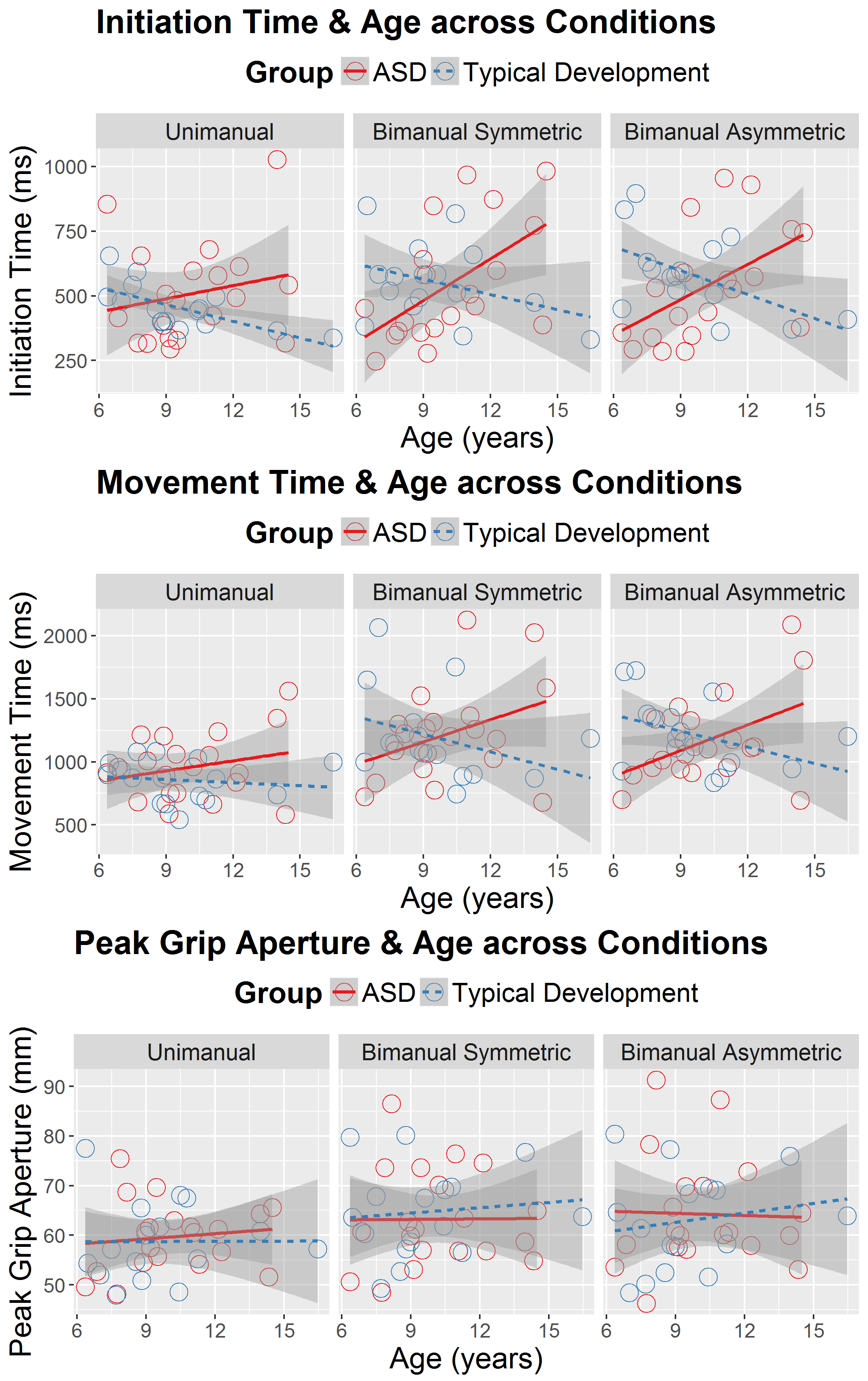


**Supplemental Figure 2**. Representative hand velocity profiles of one younger child with TD (Male 8.7 years) and one younger child with ASD (Male, 9.1 years). These profiles show that for the younger child with ASD, MTs were similar to the TD participant in the unimanual conditions, but faster in the bimanual symmetric and asymmetric conditions.

**Supplemental Figure 3**. Representative hand velocity profiles of one older child with TD (Male 10.8 years) and one older child with ASD (10.9 years). These profiles show that MTs were longer across all conditions for the older child with ASD when compared to the older child with TD.
